# A novel dual DYRK1A/B inhibitor for the treatment of type 1 diabetes

**DOI:** 10.3389/fphar.2025.1657042

**Published:** 2025-10-13

**Authors:** Šarūnas Tumas, Jonas Mingaila, Vytautas Baranauskas, Emilija Baltrukonytė, Laurynas Orla, Jan Aleksander Krasko, Roberta Pocevičiūtė, Dina Berlina, Alexei Belenky, Maria Vilenchik, Agnė Vaitkevičienė, Olga Potapova, Aurelijus Burokas

**Affiliations:** ^1^ Cureline Baltic UAB, Vilnius, Lithuania; ^2^ Department of Biological Models, Institute of Biochemistry, Life Sciences Center, Vilnius University, Vilnius, Lithuania; ^3^ Institute of Translational Health Research, Faculty of Medicine, Vilnius University, Vilnius, Lithuania; ^4^ Laboratory of Immunology, National Cancer Institute, Vilnius, Lithuania; ^5^ Felicitex therapeutics UAB, Vilnius, Lithuania

**Keywords:** type 1 diabetes, DYRK1, DYRK1A kinase, DYRK1B kinase, GTT, streptozotocin

## Abstract

**Background:**

Type 1 diabetes (T1D) is an autoimmune disease that leads to the progressive destruction of pancreatic β cells, resulting in insulin deficiency and hyperglycemia. Current treatments focus on insulin replacement, but novel therapeutic approaches targeting β cell regeneration are needed. Dual-specificity tyrosine-phosphorylation-regulated kinases 1A (DYRK1A) and 1B (DYRK1B) play key roles in cell cycle regulation and β cell proliferation.

**Methods:**

In this study, FX8474, a novel DYRK1 inhibitor, was evaluated in a streptozotocin (STZ)-induced diabetic mouse model. Mice were treated orally for 7 days, and pharmacokinetics, glucose regulation, and immune cell profiling were assessed.

**Results:**

Pharmacokinetic analysis confirmed the oral bioavailability of FX8474, and treatment was associated with improved fasted glucose levels and glucose tolerance after a 7-day treatment. Immunophenotyping indicated that FX8474 treatment increases CD4+ memory T cell populations while decreasing CD4+ effector cells, as well as restores CD8+ T cell phenotypes to levels observed in healthy mice.

**Conclusion:**

FX8474 has a modest effect on glucose regulation and immune cell composition, warranting further investigation into its potential therapeutic applications.

## 1 Introduction

Type 1 diabetes (T1D) is an autoimmune disease characterized by the destruction of insulin-producing pancreatic β cells, which then results in inadequate insulin production and hyperglycemia (Gomez-Tourino et al., 2016). Patients with T1D require constant administration of insulin, which only alleviates the symptoms (hyperglycemia) but does not solve the root cause of the disease, namely, insufficient production of insulin. Novel approaches for treating T1D, such as whole pancreas or islet transplantation, are promising and aim to eradicate the root cause of the disease (Samoylova et al., 2019), (Shapiro et al., 2000). However, they are unlikely to be a viable solution for millions of people due to the high cost and issues with histocompatibility. There is evidence that some pancreatic β cells survive in T1D patients (Pugliese, 2017). Therefore, a more cost-efficient approach could be to stimulate the *in situ* regeneration of patients’ own β cells.

Dual-specificity tyrosine-phosphorylation-regulated kinases 1A (DYRK1A) and 1B (DYRK1B) are serine/threonine kinases that play crucial roles in cell cycle regulation and cell growth (Lindberg and Meijer, 2021). DYRK1A and DYRK1B are highly conserved between mouse and human at both sequence and functional levels (Dirice et al., 2016; Shen et al., 2015). These kinases are expressed in various tissues, including the pancreas, suggesting potential roles in β-cell function (Yoshida and Yoshida, 2023). In recent years, researchers have explored the potential of DYRK1A and DYRK1B inhibitors as therapeutic agents for activating pancreatic β-cell growth and ultimately treating T1D; however, pancreatic β cell expansion was a challenge due to their low proliferative ability (Gregg et al., 2012; Meier et al., 2008). Despite these challenges, several molecules have been identified that can expand pancreatic β cells *in vitro* and *in vivo*, such as 5-IT and harmine (Dirice et al., 2016; Wang et al., 2015). These were one of the first identified molecules that stimulated the proliferation of pancreatic β cells; however, harmine lacked specificity as it inhibited the activity of multiple other kinases, such as cyclin-dependent kinases (CDKs), mitogen-activated protein kinases (MAPKs), glycogen synthase kinases (GSKs), and cell division cycle-like (CDC-like) kinases (Kumar et al., 2020). To increase specificity analogs of harmine, GNF7156 and GNF4877 were produced, which specifically inhibited DYRK1A and GSK3B activities (Liu et al., 2020; Shen et al., 2015). Both these compounds showed promising results, with an increased signal of the Ki67 proliferation marker in the pancreatic islets, lower fasting glucose levels, higher secretion of insulin, and higher glucose tolerance compared to vehicle-treated diabetic rat insulin promoter and diphtheria toxin A (RIP-DTA) mice. However, testing a selective DYRK1A/1B inhibitor is worthwhile because dual inhibition of closely related DYRK isoforms directly releases β-cell cell-cycle brakes (Dirice et al., 2016; Shen et al., 2015; Wang et al., 2015) while avoiding GSK3-mediated WNT/β-catenin activation (Henriksen and Dokken, 2006; McCubrey et al., 2014; Krausova and Korinek, 2014) and metabolic pathway disruption (Henriksen and Dokken, 2006) that can cause dedifferentiation and systemic effects, providing a cleaner, more interpretable mechanism and likely a better safety window.

In this work, we describe a novel orally available DYRK1A and DYRK1B inhibitor, FX8474. FX8474 displayed a good short-term safety profile and a modest increase in glucose tolerance in an STZ-induced diabetic mouse model. Finally, we demonstrate that STZ-induced diabetic mice have decreased peripheral CD4^+^ and CD8^+^ naive T cells and increased CD8^+^ central memory cells compared to healthy mice. FX8474 modulated CD8^+^ T cells, restoring their distribution to levels observed in healthy mice.

## 2 Materials and methods

### 2.1 Animals

Before and during the experiment, animals were kept and treated according to all active legislations. All experimental procedures conformed to Directive 2010/63/EU requirements and were approved by the Lithuanian State Food and Veterinary Service (approval number G2–208, 2022). All researchers involved in the animal study have FELASA B (or equivalent to it) certificates, allowing them to work with laboratory animals.

Seven-week-old male C57BL/6 mice were purchased from Inotiv, West Lafayette, IN. Mice were housed in an IVC system at a constant temperature (25 °C ± 1 °C), with a humidity of 50% ± 10%, in a light-controlled environment (light and dark cycle each lasting 12 h) and were provided *ad libitum* access to complete rodent food and water, unless specified otherwise. Mice were acclimatized in the animal facility for at least 1 week before the start of the experiment. Cages were changed once per week.

### 2.2 Experimental compounds

FX8474 is a small-molecule DYRK1A/B inhibitor belonging to the substituted 7-azaindole/quinoline chemical class described by Felicitex et al. [U.S. Patent No. 10,577,365 (Dreas et al.) and WO2025069008A1]. It is a substituted 7-azaindole core linked to a quinoline moiety through an amide or heteroaryl bridge, optionally bearing substituents such as halogen, alkyl, alkoxy, cyano, nitro, amino, or hydroxy groups. These compounds are selective dual DYRK1A/B inhibitors with minimal or no activity against GSK3B. The compound is a proprietary, nonclinical research compound owned by Felicitex Therapeutics Inc. Natick, MA. and was supplied as hydrochloride salt.

### 2.3 Pharmacokinetics

Pharmacokinetic evaluation of FX8474 oral administration was performed using six animals after 12 h of fasting. FX8474 was suspended in vehicle solution [20% Kolliphor HS-15, 30% PEG400, and 50% 50 mM citric acid (w/w)] and introduced via oral gavage at 10 mg/kg. Consequently, blood was collected using a sparse sampling technique from three animals at each time point. Blood was collected at 10-min, 20-min, 30-min, 1-h, 2-h, 4-h, 12-h, and 24-h time points. Blood was centrifuged at 2000 *g* for 15 min at +4 °C, and serum was collected. Serum samples were stored in −20 °C until serum extraction. Organs were collected after the last time point and kept frozen until tissue extraction.

### 2.4 Serum and tissue extraction

Serum was extracted by mixing 80 µL of extraction buffer (50% methanol, 50% acetonitrile, and 0.2% formic acid) and 20 µL of serum. The mixture was vortexed for 3 min and centrifuged at 10,000 *g* for 10 min. The supernatant was further diluted 1:5 (v/v) in extraction buffer before running LC-MS analysis. Tissues (50 mg) were first homogenized using a Beadbug 6 homogenizer in 150 µL distilled water using steel beads at 4,500 rpm. Five homogenization cycles were performed lasting 30 s, followed by a 30-s pause between cycles. An aliquot of 1.7 mL of extraction buffer was added to the homogenized sample and vortexed for 5 min. The sample was centrifuged at 10,000 *g* for 10 min. The supernatant was further diluted 1:5 (v/v) in the extraction buffer before running LC-MS analysis.

### 2.5 LC-MS analysis

Chromatography was performed using the Ultimate 3000 instrument (Thermo Fisher Scientific, Waltham, MA). Mass spectrometry was performed using the Orbitrap Q-Executive Classic instrument (Thermo Fisher Scientific, Waltham, MA).

### 2.6 Maximal dose assessment

To evaluate the maximum tolerated dose (MTD) for the FX8474 compound, mice (N = 4) were administered FX8474 *via* oral gavage once per day for 5 consecutive days at the following doses: 0, 20, 50, and 100 mg/kg. Mice were monitored throughout the experiment, and weight and food/water consumption were recorded.

### 2.7 Induction of type 1 diabetes by STZ in mice

STZ destroys insulin-producing β cells in the pancreas. The injections were administered intraperitoneally (*i.p.*) at 40 mg/kg body weight for 5 consecutive days to induce a diabetic state in the mice. Food administration was stopped 4 h prior to STZ injection, but the water was provided *ad libitum*. Food and 10% sucrose solution were provided after injections. Diabetes was confirmed 9 days after the last STZ dose by measuring the glucose levels after 6 h of fasting and comparing the glucose levels to those in healthy mice. Diabetic mice with fasted glucose levels between 160 and 300 mg/dL were used for this experiment.

### 2.8 Treatment of mice

Mice were starved for 4 h before dosing. Mice were treated once per day for 7 days with vehicle [20% Kolliphor HS-15, 30% PEG400, and 50% 50 mM citric acid (w/w)], insulin (Lantus, Sanofi, 2 IU/kg), or FX8474 (10 mg/kg). The FX8474 compound was suspended in vehicle solution. The efficacy experiment consisted of three parts: a 7-day dosing period, a 7-day washout period, and a second 7-day treatment period. The intraperitoneal glucose tolerance test (ipGTT) analysis was performed after each part.

### 2.9 Glucose measurement and glucose tolerance test

As previously described (Burokas et al., 2018), mice were placed into clean cages with no food except water provided *ad libitum* 12 h before the ipGTT. After mice were fasted for 12 h, weight measurements and baseline glucose levels were recorded using a glucometer (Glucometer Contour Plus ELITE, Switzerland). Animals were injected with glucose 2 g/kg *i. p.*, and blood glucose levels were measured after 15, 30, 45, 60, 120, 180, and 240 min. Glucometer results were recorded for each mouse at each time point. After the last measurement was taken, mice were given food *ad libitum.*

### 2.10 Preparation of formalin-fixed paraffin-embedded (FFPE) blocks

Pancreatic tissue specimens were fixed in 10% neutral buffered formalin (Sigma-Aldrich, Burlington, MA) at room temperature for 16 h, dehydrated through a graded ethanol series (70%, 80%, 95%, and 100%; VWR Chemicals, Radnor, PA), cleared in xylene (Merck, Darmstadt, Germany), and infiltrated with paraffin wax (Paraplast X-tra, Sigma-Aldrich, Burlington, MA) using a standard automated tissue processor. Tissues were embedded in fresh molten paraffin using stainless steel molds and cooled on a cold plate. Paraffin blocks were sectioned at 5 µm thickness on a rotary microtome (Leica Biosystems, Nussloch, Germany), and sections were mounted on Superfrost Plus adhesive slides (Thermo Fisher Scientific, Waltham, MA) for subsequent histological analysis.

### 2.11 Immunohistochemistry

Paraffin-embedded pancreatic sections were deparaffinized, rehydrated, and subjected to antigen retrieval in 10 mM citrate buffer (pH 6.0) at 95 °C for 20 min. Next, tissues were blocked with 5% normal goat serum in PBS +0.1% Triton X-100 for 1 h. Incubation with the guinea pig anti-insulin antibody (1:200, Abcam, Cambridge, United Kingdom) primary antibody was carried out overnight at 4 °C. Slides were washed three times with PBS and incubated with the HRP-conjugated goat anti-guinea pig secondary antibody (1:500, Jackson Immunoresearch laboratories, West Grove, PA) for 1 h at room temperature. The signal was developed with the DAB substrate (Vector Laboratories, Newark, CA), counterstained with hematoxylin, dehydrated, and mounted. Images were acquired using a Leica Biosystems, Nussloch, Germany DM2000 microscope.

### 2.12 Insulin analysis

Blood was collected on the second day after the first treatment cycle (during the washout period). The EDTA-containing Microvette^®^ tubes containing blood samples were centrifuged at 2000 *g* for 15 min at 4 °C to obtain plasma fraction for the analyses of insulin levels. The EMINS mouse ELISA kit (Thermo Fisher Scientific, Waltham, MA) was used to quantify insulin. Measurements were performed according to the manufacturer’s instructions.

### 2.13 Immunophenotyping

Blood (100 µL) was collected on the second day after the first treatment cycle (during the washout period). Samples were processed sequentially through erythrocyte lysis, Fc blocking, and antibody staining. For erythrocyte lysis, 900 µL of distilled water was added, and the sample was vortexed for 3 s, after which 100 µL of 10 x PBS (Corning Inc., Corning, NY, #46–013-CM) was added to restore a final concentration of 1 x PBS. Cells were washed once with 1 x PBS and were resuspended in 100 µL of stain buffer (Becton, Dickinson and Company (BD), Franklin Lakes, NJ #554657). Fc receptors were blocked at RT for 10 min, according to the manufacturer’s instruction (Becton, Dickinson and Company (BD), Franklin Lakes, NJ #553142). Without a washing step, antibodies were added for surface staining at +4 °C for 20 min: anti-CD3-FITC (Becton, Dickinson and Company (BD), Franklin Lakes, NJ #557666), anti-CD44-APC (Becton, Dickinson and Company (BD), Franklin Lakes, NJ #559250), anti-CD19-PerCP-Cy5.5 (Becton, Dickinson and Company (BD), Franklin Lakes, NJ #551001), anti-NK1.1-PE (Becton, Dickinson and Company (BD), Franklin Lakes, NJ #557391), anti-CD62L-BV421 (Becton, Dickinson and Company (BD), Franklin Lakes, NJ #56910), anti-CD8-BV500 (Becton, Dickinson and Company (BD), Franklin Lakes, NJ #560776), anti-CD44-PE-Cy7 (Becton, Dickinson and Company (BD), Franklin Lakes, NJ #552775), and anti-CD45-APC-Cy7 (Becton, Dickinson and Company (BD), Franklin Lakes, NJ #557659). Cells were washed twice with stain buffer before acquisition on an LSR II cytometer (Becton, Dickinson and Company (BD), Franklin Lakes, NJ). Analysis was performed using FlowJo software (Becton, Dickinson and Company (BD), Franklin Lakes, NJ). The gating strategy is presented in Supplementary Figure S4.

## 3 Results

### 3.1 Pharmacokinetic profile of the DYRK1 inhibitor FX8474

**FIGURE 1:**
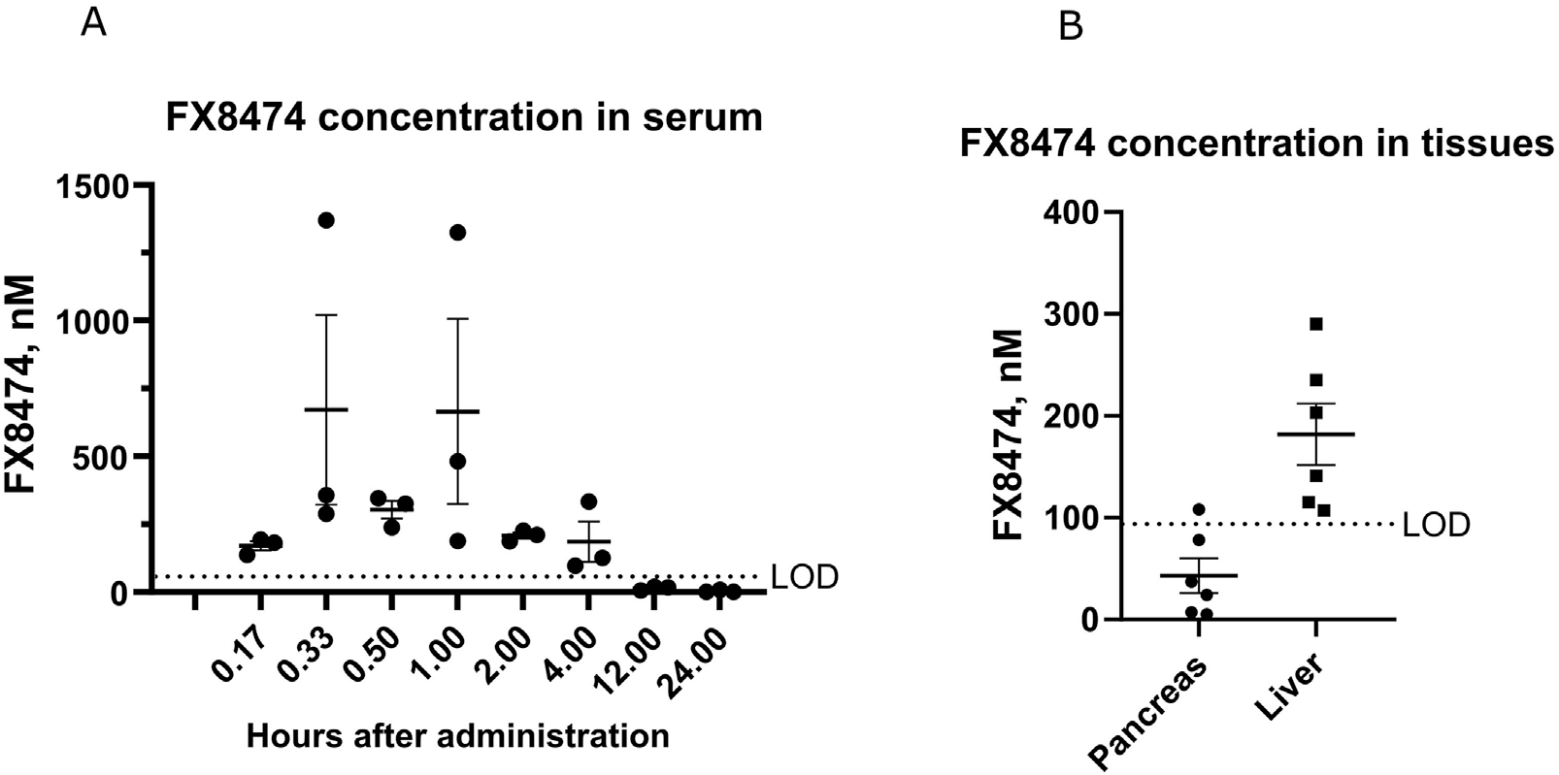
Pharmacokinetic results for FX8474 after oral administration. Animals received a single oral dose of FX8474 at 10 mg/kg after 12 h of fasting. **(A)** FX8474 levels in serum. **(B)** FX8474 levels in the pancreas and liver after 24 h. Individual values, averages, and SEM are shown from three animals at each time point. LOD, limit of detection.

The pharmacokinetic profile of orally administered FX8474 was determined with a single oral 10-mg/kg dose, and serum was collected using the sparse sampling technique from three animals at each time point. Detected FX8474 serum levels reached the maximum concentration up to 700 nM in serum and were cleared from the body within 12 h after administration (Figure 1A). FX8474 was detected in the liver (100–300 nM) but not in pancreatic tissues (most samples were below LOD) after 24 h (Figure 1B).

### 3.2 Maximum tolerated dose of FX8474

**FIGURE 2:**
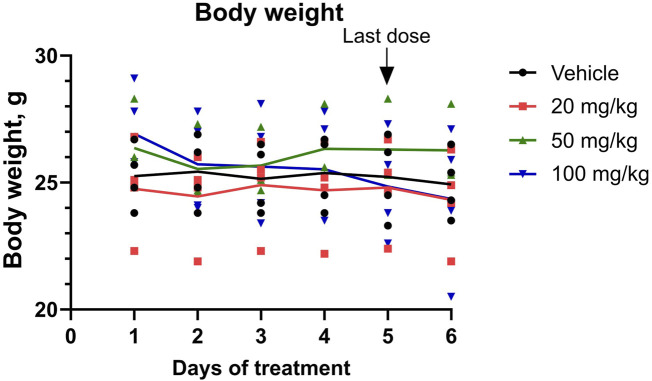
Mouse body weight during the MTD experiment. Mice received daily oral administration of FX8474 at 20, 50, or 100 mg/kg for 5 consecutive days to determine the maximum tolerated dose (MTD). Individual values for each mouse is shown. Connected lines indicate the mean for each group, N = 4. Two-way ANOVA with Tukey’s multiple comparison test was performed to determine statistical significance. There were no statistically significant differences between treatment groups.

To identify whether repeated short-term dosing is safe for the animals, an MTD of FX8474 was determined by administering FX8474 orally once per day for 5 days at 0, 20, 50, and 100 mg/kg/day. The condition of the mice was monitored throughout the experiment. No short-term toxic effects were observed based on visual inspection or changes in mouse body weight (Figure 2). There were no differences in food or water consumption between groups (data not shown). One mouse died in the 50-mg/kg group on day 4; however, no adverse clinical signs were observed before loss of the animals, and no pathological changes were detected during gross necropsy.

### 3.3 Drug efficacy study in an STZ-induced type 1 diabetes model

**FIGURE 3:**
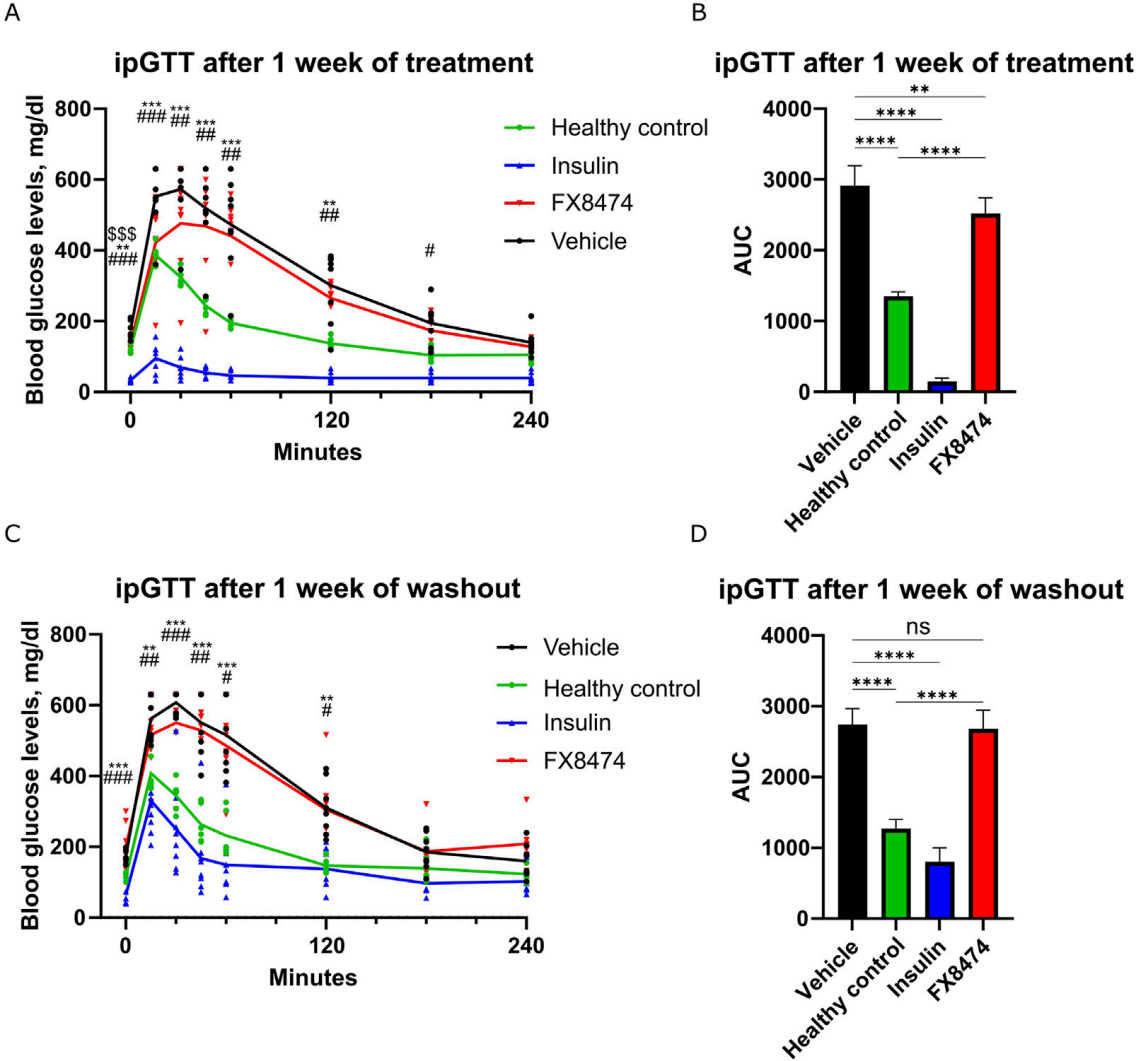
ipGTT after the first cycle of treatment and washout. Mice were treated once per day for 7 days with vehicle, insulin (2 IU/kg), or FX8474 (10 mg/kg). **(A)** ipGTT after 7 days of daily treatment. **(B)** Area under the curve (AUC) analysis of the ipGTT profile from **(A)**. **(C)** ipGTT was performed after a week of washout. **(D)** AUC analysis of the ipGTT profile from **(C)**. Mean values and standard deviation are plotted. For ipGTT results, two-way ANOVA with Dunnett’s multiple comparison test was performed to determine statistical significance compared to vehicle-treated mice. *p < 0.05, **p < 0.01, and ***p < 0.001 vs. healthy control; #p < 0.05, ##p < 0.01, and ###p < 0.001 vs. insulin group; $$$ p < 0.001 vs. FX8474. For AUC, statistical significance was determined using one-way ANOVA with Tukey’s multiple comparisons test. ns, not significant *p < 0.05; **p < 0.01; ***p < 0.001. N = 6 for healthy control and N = 8 other groups. Only statistically significant differences are shown.

We hypothesized that after induction of severe diabetes, the surviving pancreatic beta-cell mass would be too small to be regenerated, but a less severe disease phenotype might be more susceptible for therapy. Therefore, we used STZ-induced diabetic mice with moderate diabetic disease state (fasting glucose levels of 160–300 mg/dL). These mice have reduced insulin staining within their pancreatic islets (Supplementary Figure S1). Next, we tested the drug efficacy of the DYRK1 inhibitor FX8474. Mice were allocated to treatment groups based on their fasting glucose levels (Supplementary Figure S2A). The mice were orally administered vehicle, insulin, or FX8474 once per day for 7 days. ipGTTs were carried out after 1 week of daily dosing (first cycle of treatment) and after a 1-week washout period (washout). Mice treated with FX8474 had reduced fasted glucose levels compared to vehicle-treated mice (Supplementary Figure S2B **).** Mice treated with FX8474 showed better ipGTT profiles than vehicle-treated mice, along with significantly lower values of area under the curve (AUC, Figures 3A,B). Insulin levels were similar between FX8474 and vehicle-treated mice (Supplementary Figure S3). After 1 week of washout, FX8474-treated mice had similar fasted glucose levels and glucose tolerance to vehicle-treated mice (Supplementary Figure S2C; Figures 3C, D).

**FIGURE 4:**
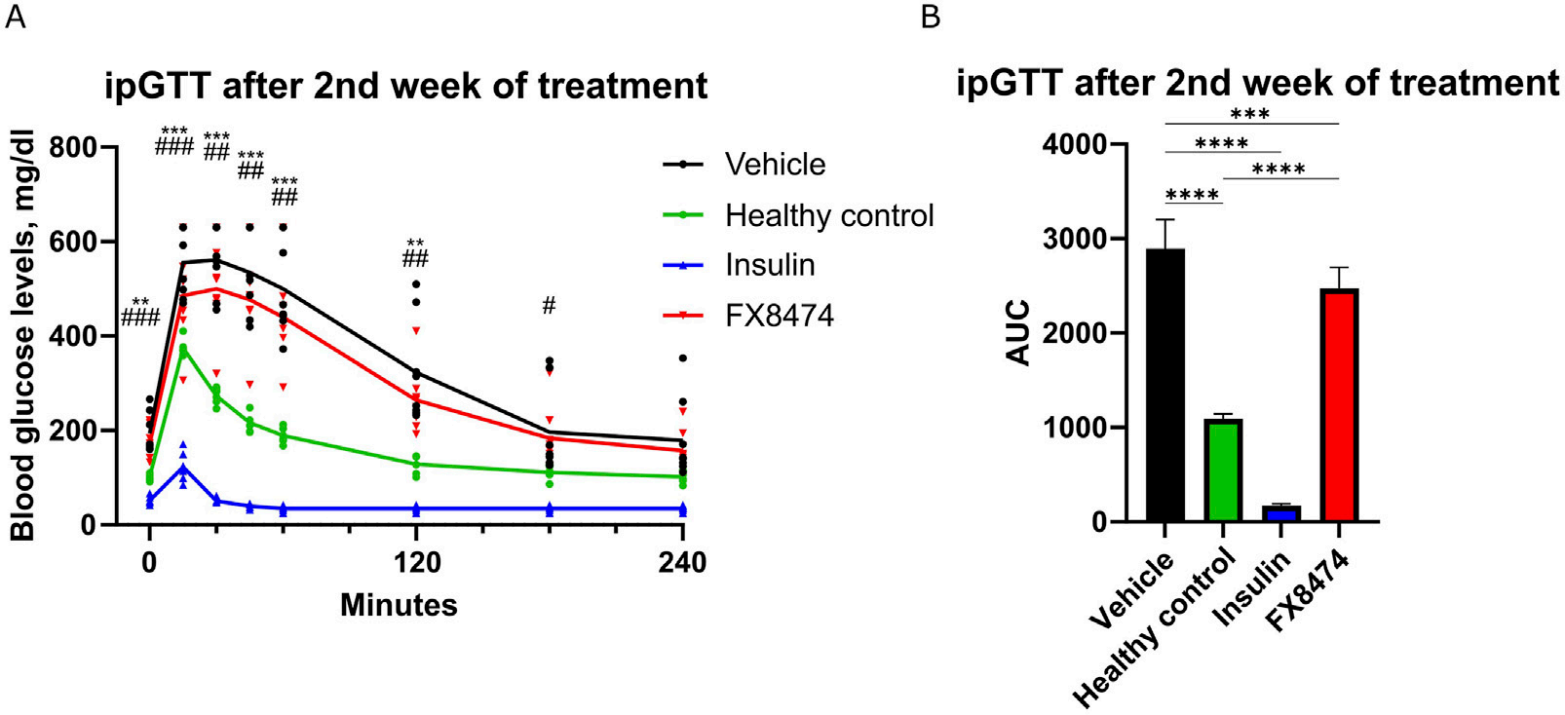
ipGTT after the second cycle of treatment. Mice were treated once per day for 7 days with vehicle, insulin (2 IU/kg), or FX8474 (10 mg/kg). **(A)** ipGTT after 7 days of daily treatment. **(B)** AUC analysis of the ipGTT profile from **(A)**. Mean values and standard deviation are plotted. For ipGTT results, two-way ANOVA with Dunnett’s multiple comparison test was performed to determine statistical significance compared to vehicle-treated mice. *p < 0.05, **p < 0.01, and ***p < 0.001 vs. healthy control; #p < 0.05, ##p < 0.01, and ###p < 0.001 vs. insulin group. For AUC, statistical significance was determined using one-way ANOVA with Tukey’s multiple comparisons tests. *p < 0.05; **p < 0.01; ***p < 0.001. N = 8 for vehicle, N = 6 for healthy control, and N = 8 for other groups. Only statistically significant differences are shown.

After the washout period, mice received a second cycle of treatment for 1 week. Similar to earlier results, FX8474-treated mice showed similar fasted glucose levels to vehicle-treated mice but had better glucose tolerance (Supplementary Figure S2D; Figures 4A, B).

**FIGURE 5:**
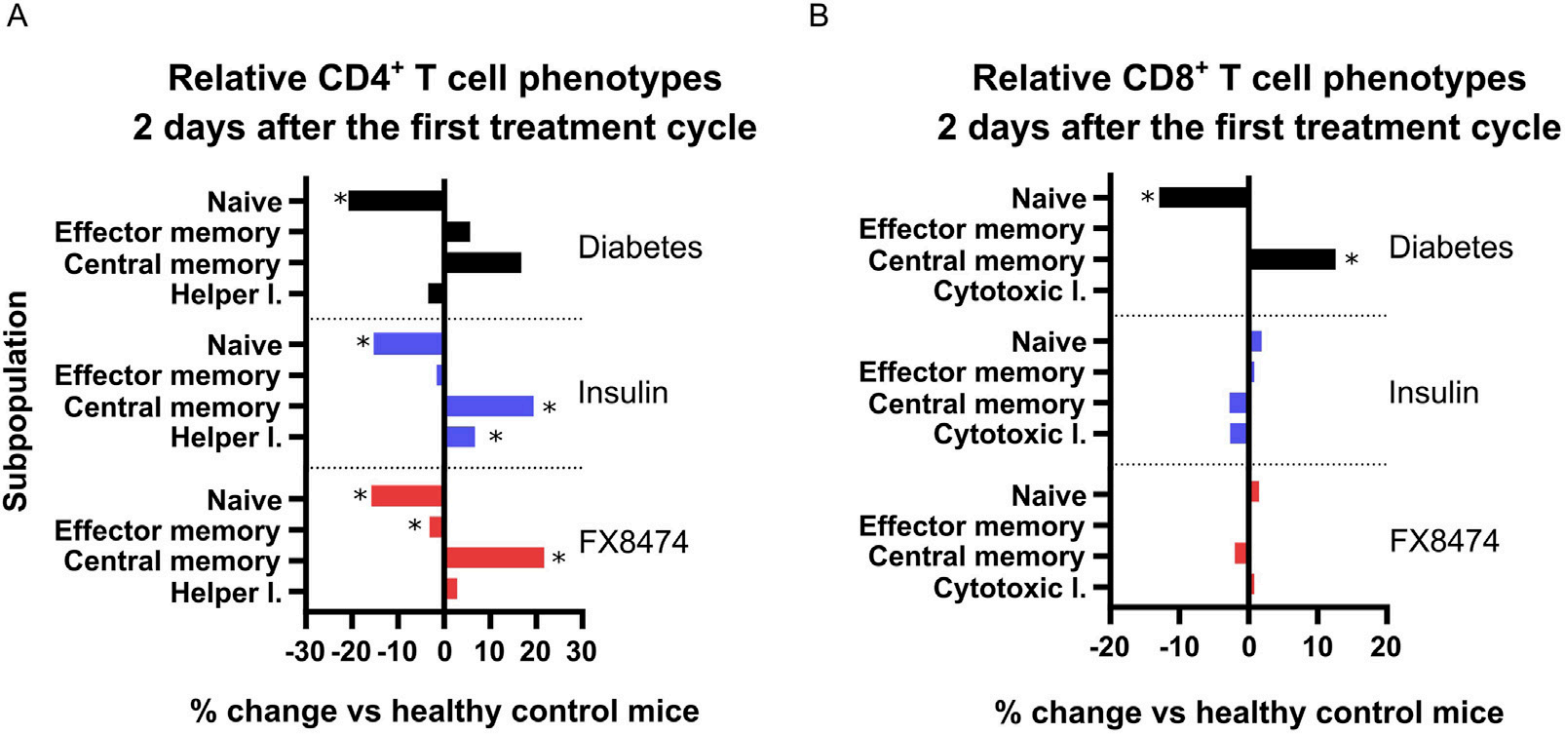
FX8474 treatment has different effects on CD4^+^ and CD8^+^ T-cell phenotypes. Lymphocytes from fresh whole blood were collected 2 days after the end of the first round of treatment and were analyzed using flow cytometry to assess CD4^+^ **(A)** and CD8^+^ **(B)** T-cell subpopulations. Mean is shown. N = 6. Statistical significance was determined using one-way ANOVA with Tukey’s multiple comparison tests. *p < 0.05.

Treatment groups show distinct differences in CD4^+^ T-cell subpopulations but not in CD8^+^ T-cell phenotypes. Fresh whole blood was used to perform immunophenotyping analysis 2 days after the end of the first treatment cycle (Supplementary Figure S5). Vehicle-treated mice showed higher percentages of B cells, but there were no differences in NK or T-cell numbers between groups (Supplementary Figure S5A). There was a small increase in the CD4^+^/CD8^+^ ratio in the insulin-treated group (Supplementary Figure S5B). Immunophenotyping revealed interesting changes in T-cell composition in the blood when compared to that in the healthy control group (Supplementary Figure S5C; Figure 5). Mice in the diabetes group showed a decrease in naïve CD4^+^ T cell population, and there was a slight, but not significant, increase in those of effector and memory cells (Figure 5A). Insulin-treated mice reflected the same trend as the diabetes group. Mice treated with FX8474 showed an increase in CD4^+^ central memory cells (similar to insulin-treated mice) but had a slight decrease in CD4^+^ effector memory cells.

Regarding CD8^+^ T cells, mice in the diabetes group showed an increase in central memory cells and a decrease in naïve cells. Both insulin and FX8474 rescued the CD8^+^ T-cell phenotype, restoring their distribution to levels observed in healthy mice (Figure 5B).

## 4 Discussion

The results of this study suggest that DYRK1 inhibition has a role in modulating glucose homeostasis and immune responses in T1D. Previous research has demonstrated that DYRK1A/B inhibitors, such as harmine, can induce β-cell proliferation, yet their therapeutic applicability remains limited due to nonspecific kinase inhibition and associated toxicity (Dirice et al., 2016), (Wang et al., 2015). Our findings indicate that FX8474, a DYRK1-specific inhibitor, may offer a more targeted approach, although the observed effects were modest.

Pharmacokinetic analysis confirmed that FX8474 is orally bioavailable, reaching measurable concentrations in the serum, and is cleared within 12 h (Figure 1A) but persists in the liver at 200 nM after 24 h (Figure 1B), suggesting hepatic accumulation due to transporter-mediated uptake, lipophilicity, or slow metabolism. This raises concerns about off-target effects or hepatotoxicity. Future studies should perform a thorough investigation of longer-term toxicity, including histological examination of the liver, and assess hepatic transporters, metabolite formation, and subcellular localization and translocation through the blood–brain barrier. Nevertheless, FX8474 was safe up to doses of 100 mg/kg for 5-day exposure upon repeated daily administration (Figure 2).

We demonstrate that FX8474 is orally bioavailable and has a positive, albeit modest, anti-diabetic effect. The treatment significantly lowered fasting glucose levels, but insulin levels were not changed compared to those in vehicle-treated mice (Supplementary Figures S2, S3). Glucose tolerance was improved following FX8474 administration (Figures 3, 4), suggesting partial functional recovery of insulin secretion. Although ipGTT results suggest that the increase in glucose tolerance is due to an increase in the pancreatic β-cell count, immunohistochemical evaluation of insulin and β-cell growth markers should be investigated.

T1D progression is linked to the autoreactive immune cells, particularly CD8^+^ cytotoxic T cells, which directly contribute to the destruction of β cells (Clark et al., 2017). Our immunophenotyping analysis revealed an increase in CD8^+^ central memory T cells and decrease in naïve T cells in diabetic mice, which indicates an inflammatory response, likely due to STZ insult and pancreatic β-cell destruction. This phenotype was rescued in the insulin and FX8474 groups, which restored all CD8^+^ T-cell levels to those resembling healthy mice. This is consistent with previous findings that insulin therapy can normalize CD8^+^ T-cell profiles in a rat STZ model in the liver by reducing antigen load and hyperglycemia-associated inflammation (Lee et al., 2016). The rescue of CD8^+^ T-cell phenotypes to healthy levels in both insulin and FX8474 groups suggests a potential normalization of immune homeostasis, which may reduce inflammation and promote a more balanced immune environment under diabetic conditions. The slight decrease in CD4^+^ effector memory cells in the FX8474 group could indicate a dampening of active immune responses, although the functional consequences remain unclear. Similar findings have been reported in a T1D intervention model, where tolerogenic vaccination significantly reduced CD4^+^ effector memory T cells and increased central memory cells in the blood and pancreas of treated diabetic mice, indicating a shift toward a less activated immune state (Zhang, et al., 2013). Nevertheless, further research is needed to determine whether these immunological changes are beneficial in the context of β-cell restoration.

There are some important aspects that need to be mentioned about our study before aligning with other studies that used DYRK1 inhibitors. For example, Shen et al used *i.p.* dosing in immune-compromised animals transplanted with human pancreatic β cells (Shen et al., 2015). Although this is a good model for assessing human pancreatic β-cell proliferation, it is performed in an immunosuppressed host. The immune system is very important for the development and progression of T1D; thus, it is important to also study it in an immune-competent host. In addition, *i.p.* dosing, although advantageous as it can allow higher systemic doses to bypass oral availability, presents issues in the clinic with patient compliance. A more comparable study by Liu et al. (2020) reported beneficial outcomes on glucose tolerance in the RIP-DTA mouse model after 35 days of oral dosing (Liu et al., 2020). We have observed a beneficial effect after only 7 days of oral dosing, indicating that optimization of dosing and dosing duration should be investigated further.

Despite these observations, several limitations must be considered. The STZ model primarily induces β-cell loss through chemical toxicity rather than mimicking the autoimmune nature of human T1D. Although there are reports that STZ can induce autoantibodies against insulin (Wright et al., 2024), future studies should incorporate autoimmune-prone models, such as NOD mice, to better evaluate the long-term immunological effects of DYRK1 inhibition. Although DYRK1 inhibition is expected to promote β-cell expansion, addressing the underlying autoimmune component may require combination therapy. Notably, increased pancreatic fibrosis has been observed in T1D patients (Wright et al., 2024) and STZ-induced models (Hwang et al., 2015). Certain kinases, such as ROCK2, play key roles in fibrosis by driving IL-17–mediated inflammation (Nalkurthi et al., 2022), (Tan et al., 2013), and IL-17 knockout in STZ-treated Akita mice results in milder diabetes (Qiu et al., 2021). Therefore, targeting IL-17 signaling through ROCK2 inhibition, alongside DYRK1 inhibition, may provide a more comprehensive therapeutic approach. Given the established links between metabolic dysfunction, immune perturbations, and cognitive decline under diabetes-related conditions (Burokas and Mela, 2025), future studies should also evaluate whether targeted pharmacological modulation of kinases such as DYRK1A/B may offer parallel benefits in preserving cognitive function while maintaining metabolic control.

## 5 Conclusion

This study suggests that the DYRK1 inhibitor FX8474 may have a modest effect on glucose tolerance and immune cell populations in a diabetic mouse model. Fasting glucose levels were reduced after 1 week of treatment, but the observed changes in glucose tolerance and immune cell composition warrant further research. FX8474-treated mice showed an increase in CD4^+^ memory T cells, while CD8^+^ T-cell phenotypes were restored to levels observed in healthy mice. This suggests a possible shift in immune activity. Future studies should focus on long-term efficacy, autoimmune models, and potential combinatorial strategies with other therapeutic agents. If successfully validated, DYRK1 inhibition could represent a complementary approach in the broader landscape of T1D treatment.

## Data Availability

The original contributions presented in the study are included in the article/Supplementary Material; further inquiries can be directed to the corresponding author.

## Abbreviations

CDC-like, cell division cycle-like; CDK, cyclin-dependent kinases; DYRK1A, dual-specificity tyrosine-phosphorylation-regulated kinase 1A; DYRK1B, dual-specificity tyrosine-phosphorylation-regulated kinase 1B; GSK, glycogen synthase kinase; ipGTT, intraperitoneal glucose tolerance test; MAPK, mitogen-activated protein kinase; STZ, streptozotocin; T1D, type 1 diabetes.
