# Supplementary figures and images for: A novel dual DYRK1A/B inhibitor for the treatment of type 1 diabetes
Source: Front Pharmacol. 2025 Oct 13;16:1657042. doi: 10.3389/fphar.2025.1657042 (PMC12555008; doi:10.3389/fphar.2025.1657042)

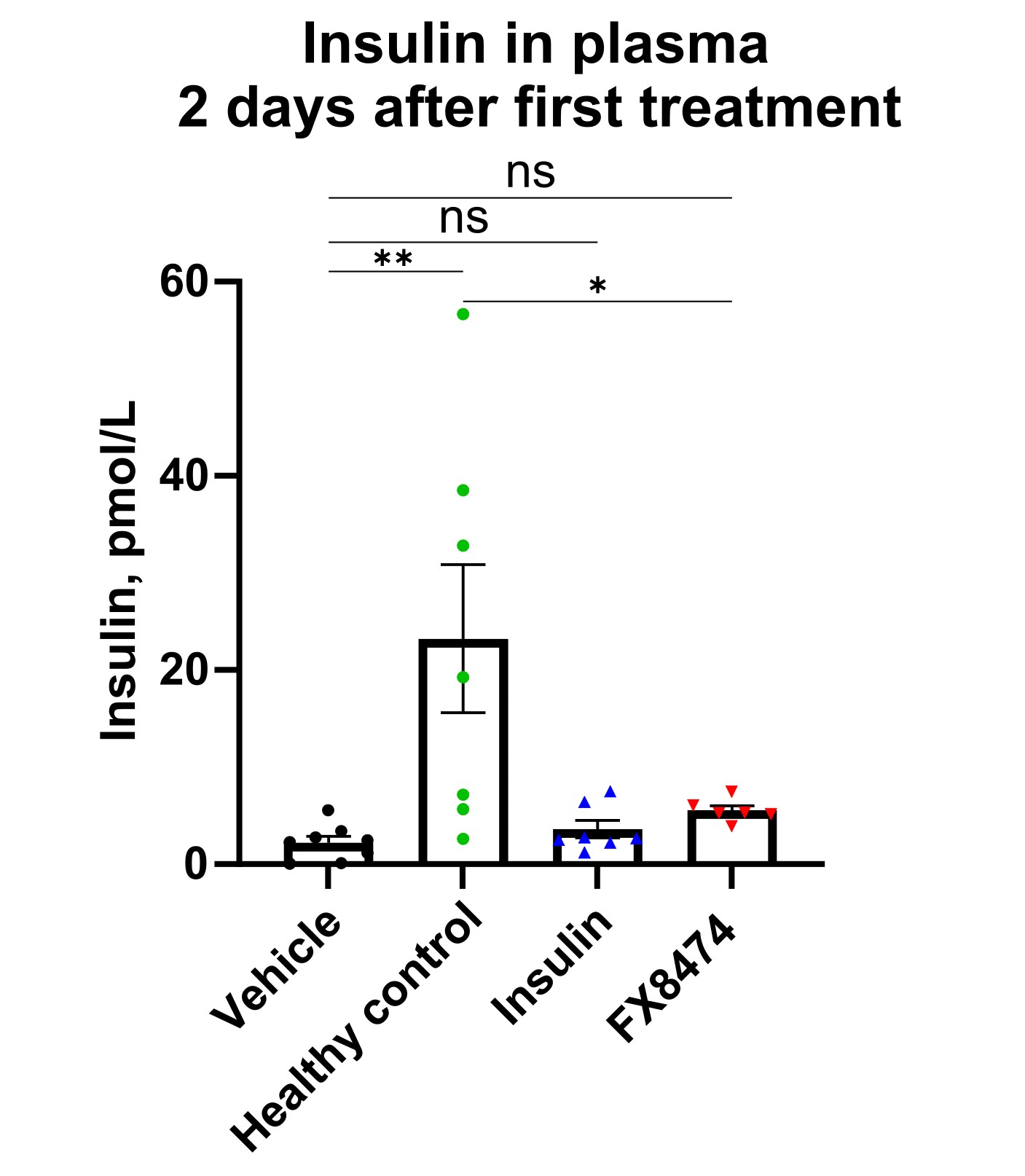

Supplement: Supplementary file 1 [file Image3.jpeg]

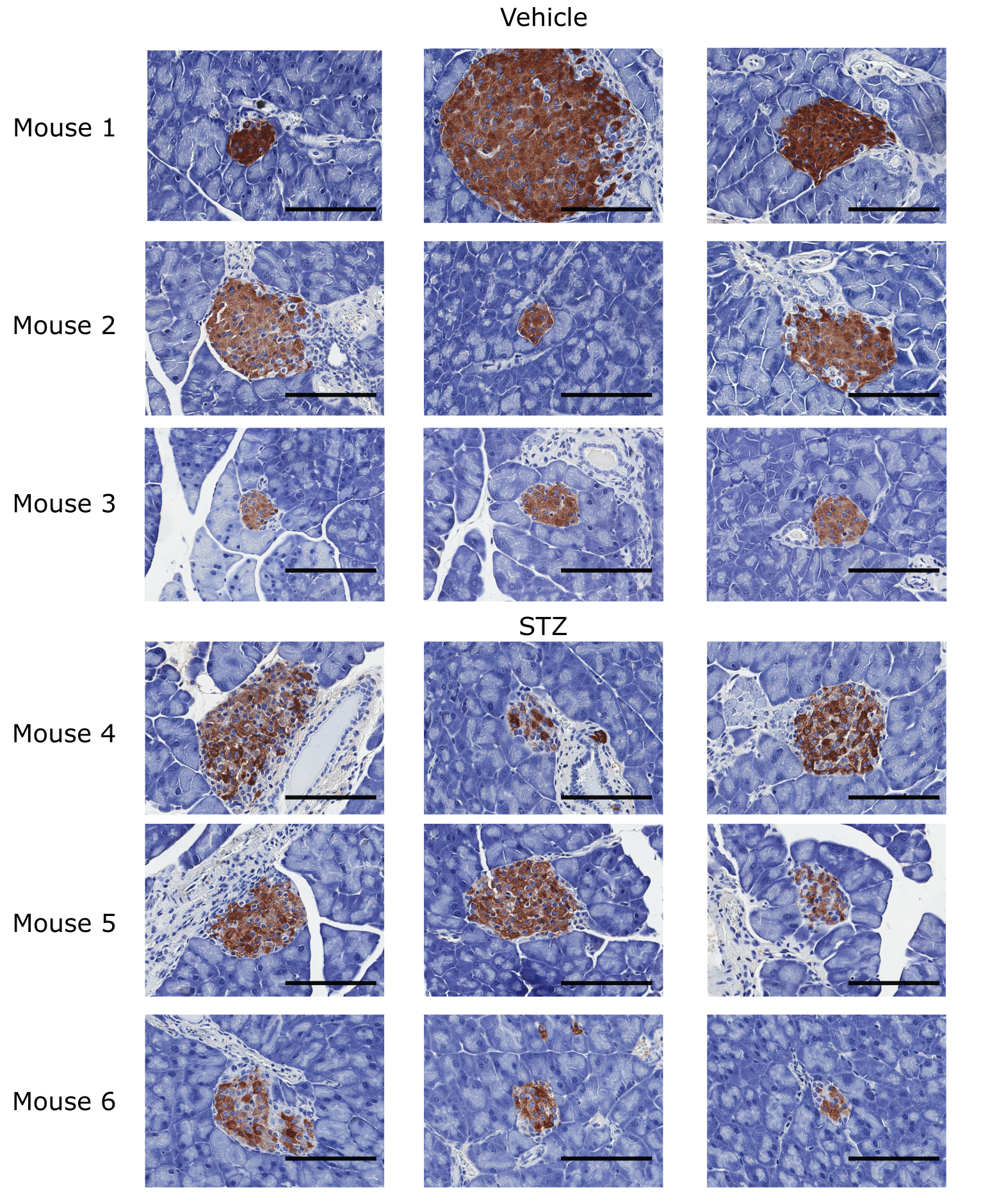

Supplement: Supplementary file 2 [file Image1.tiff]

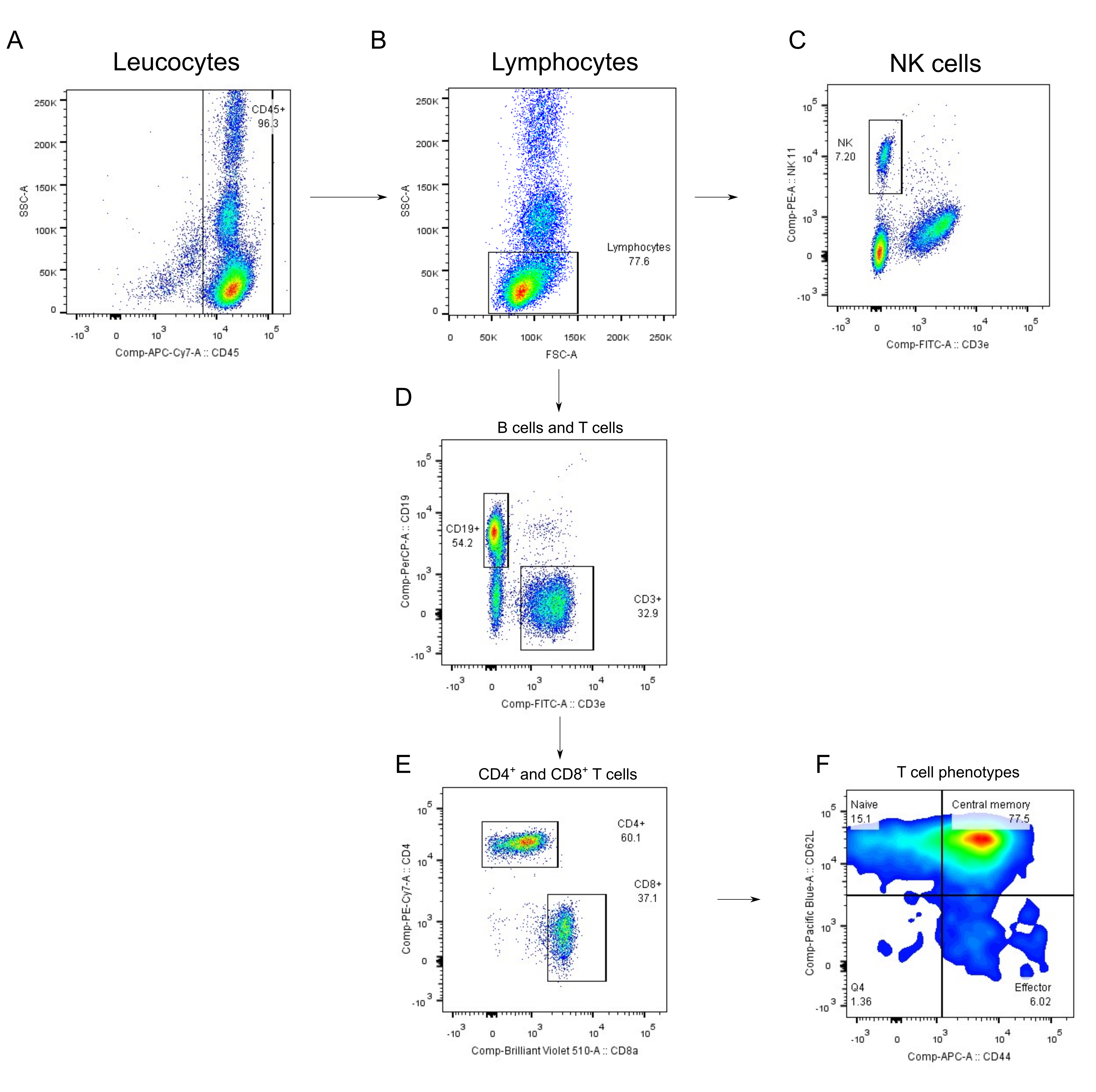

Supplement: Supplementary file 4 [file Image4.jpeg]

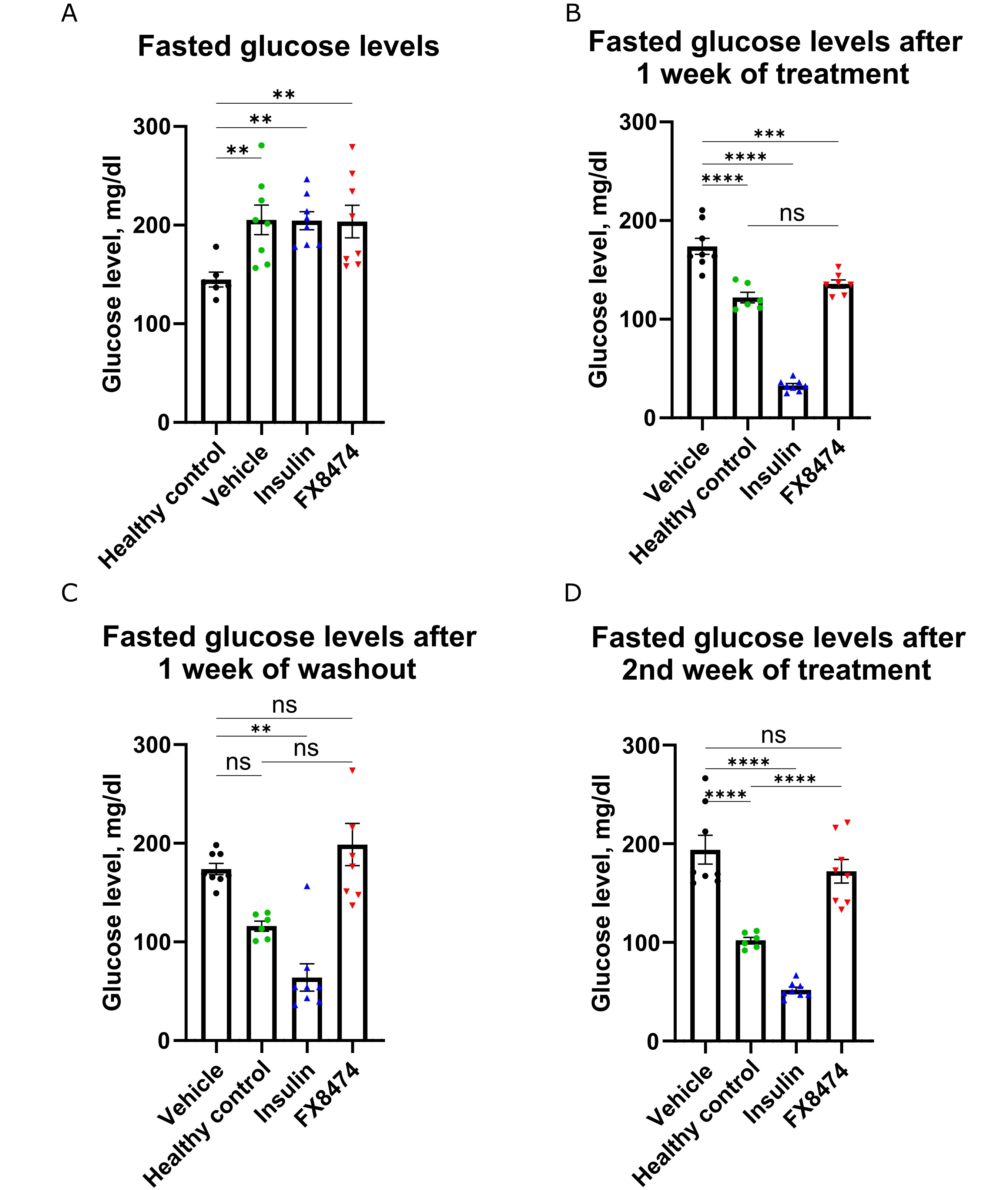

Supplement: Supplementary file 5 [file Image2.jpeg]

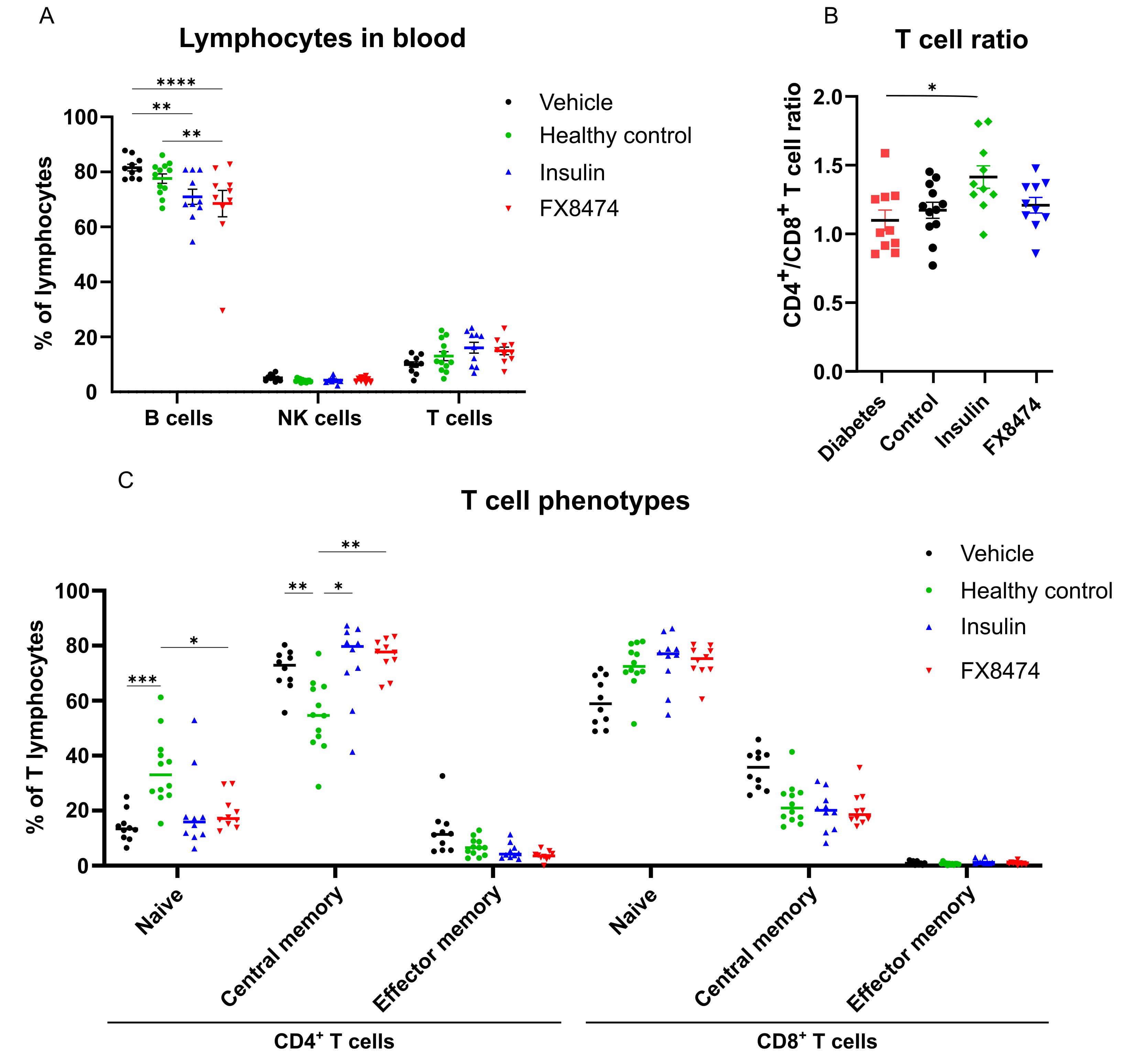

Supplement: Supplementary file 6 [file Image5.jpeg]
