# Supplementary material for: A novel dual DYRK1A/B inhibitor for the treatment of type 1 diabetes
Source: Front Pharmacol. 2025 Oct 13;16:1657042. doi: 10.3389/fphar.2025.1657042 (PMC12555008; doi:10.3389/fphar.2025.1657042)

**A Novel dual DYRK1A/B Inhibitor for the treatment of Type 1 Diabetes**

Šarūnas Tumas^1#^, Jonas Mingaila^2#^, Vytautas Baranauskas^1,3^, Emilija Baltrukonytė^2^, Laurynas Orla^1^, Jan Aleksander Krasko^4^, Roberta Pocevičiūtė^1^, Dina Berlina^1^, Alexei Belenky^5^, Maria Vilenchik^5^, Agnė Vaitkevičienė^1^, Olga Potapova^1^, Aurelijus Burokas^2^

^1^Cureline Baltic UAB, Vilnius, Lithuania.

^2^Department of Biological Models, Institute of Biochemistry, Life Sciences Center, Vilnius University, Vilnius, Lithuania.

^3^Institute of Translational Health Research, Faculty of Medicine, Vilnius University, Vilnius, Lithuania.

^4^Laboratory of Immunology, National Cancer Institute, Vilnius, Lithuania.

^5^Felicitex therapeutics UAB, Vilnius, Lithuania.

**Supplementary Material**

**
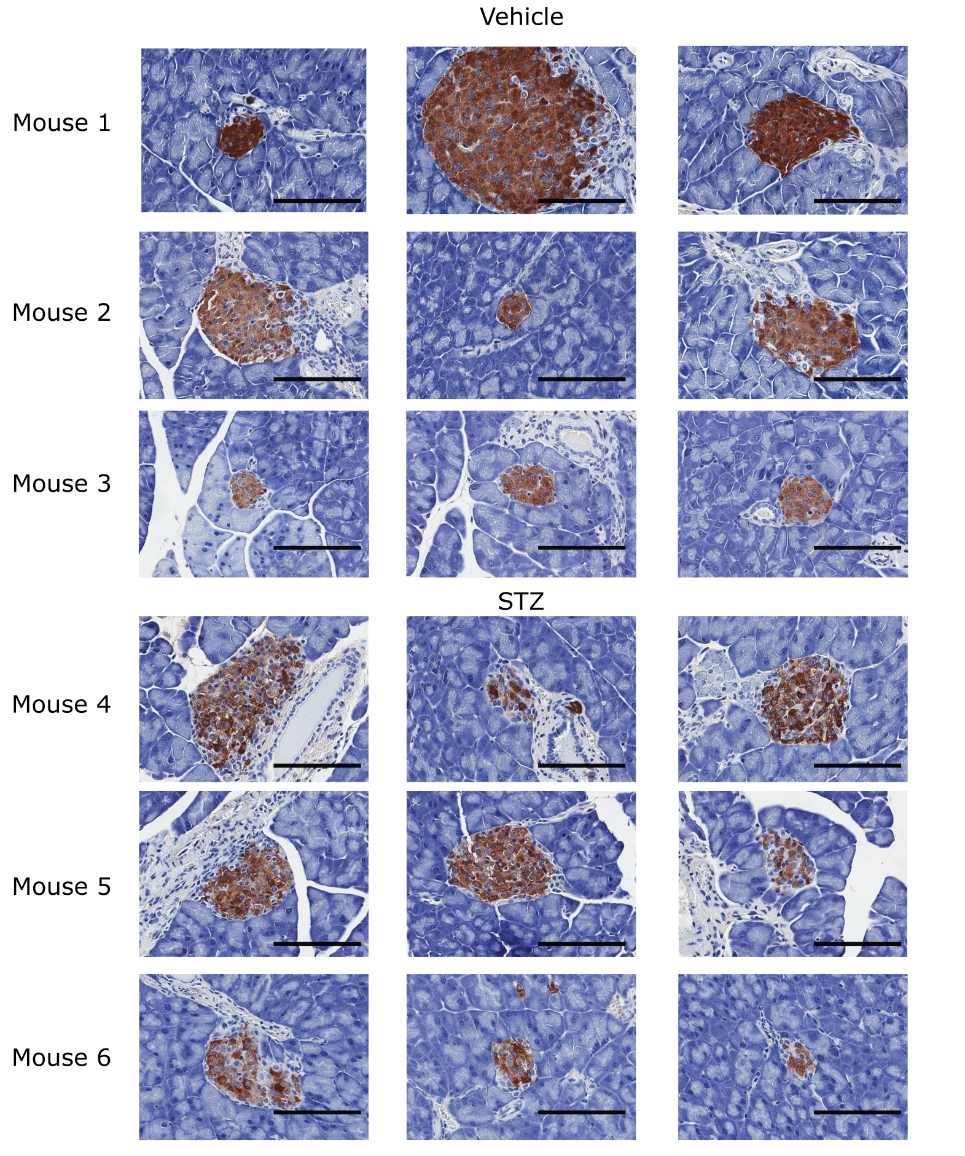
**

| **Figure S1. Diabetic mice show reduced staining with insulin.** Insulin IHC images from vehicle treated (Mouse 1-3) and STZ treated mice (Mouse 4-6). Three representative pictures are shown from each mouse. |
| --- |

**
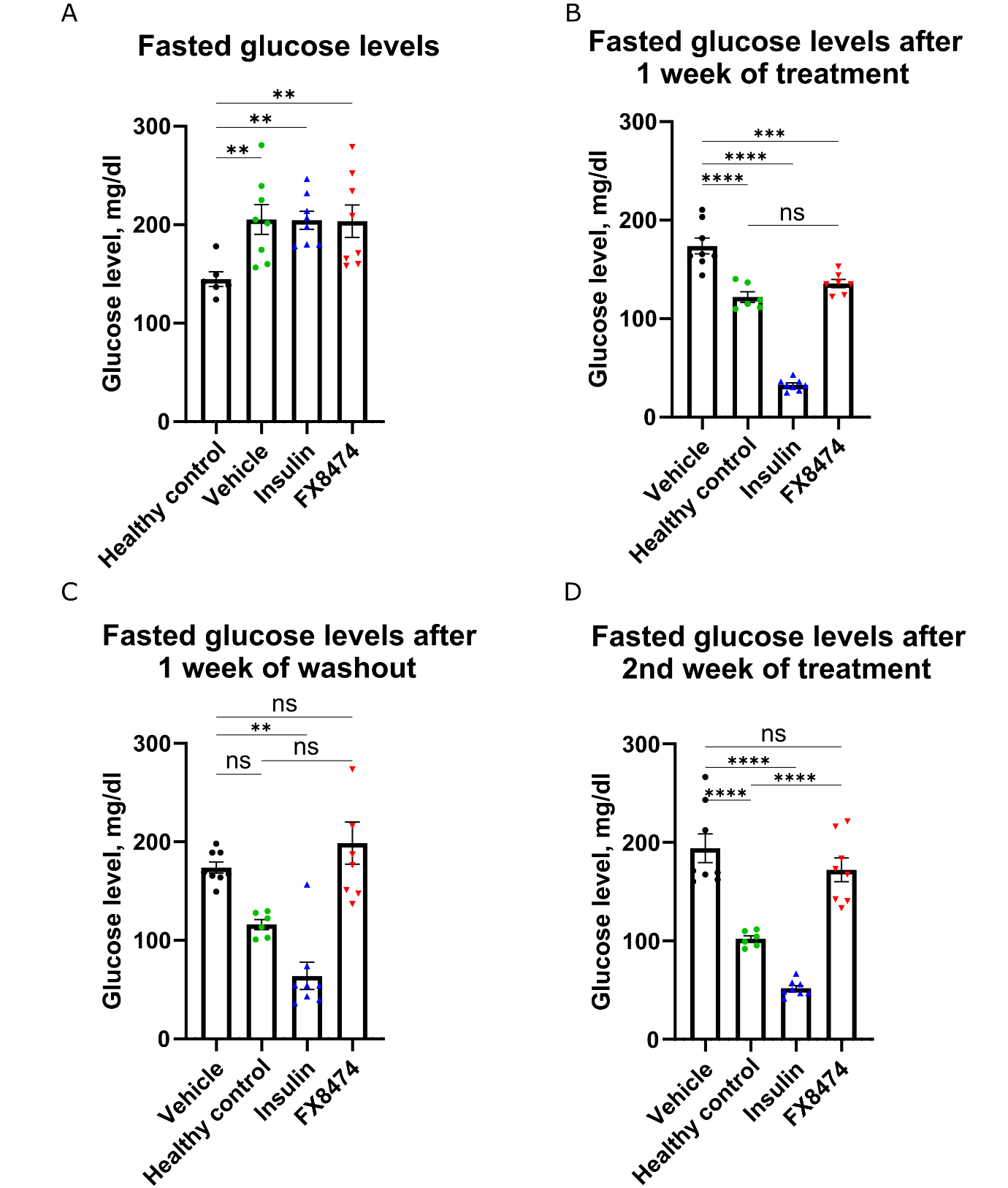
**

**Figure S2. Fasted glucose levels after STZ-induced diabetes and after treatment with FX8474.** **A)** Nine days after the last STZ dose, mice were fasted for 6 hours and glucose levels were measured. Mice were allocated to treatment groups based on their fasted glucose levels. Before ipGTT mice were fasted for 12 hours and the fasted glucose levels were measured. **B)** Fasted glucose levels after the first week of treatment. **C)** Fasted glucose levels after one week of washout. All mice were not treated, except the insulin group. **D)** Fasted glucose levels after the second week of treatment. One-way ANOVA was performed to determine statistical significance. Asterisks indicate statistical significance compared to vehicle-treated group. ** p<0,01; **** p<0,0001. Mean values with SD are plotted. N=6-8.

**
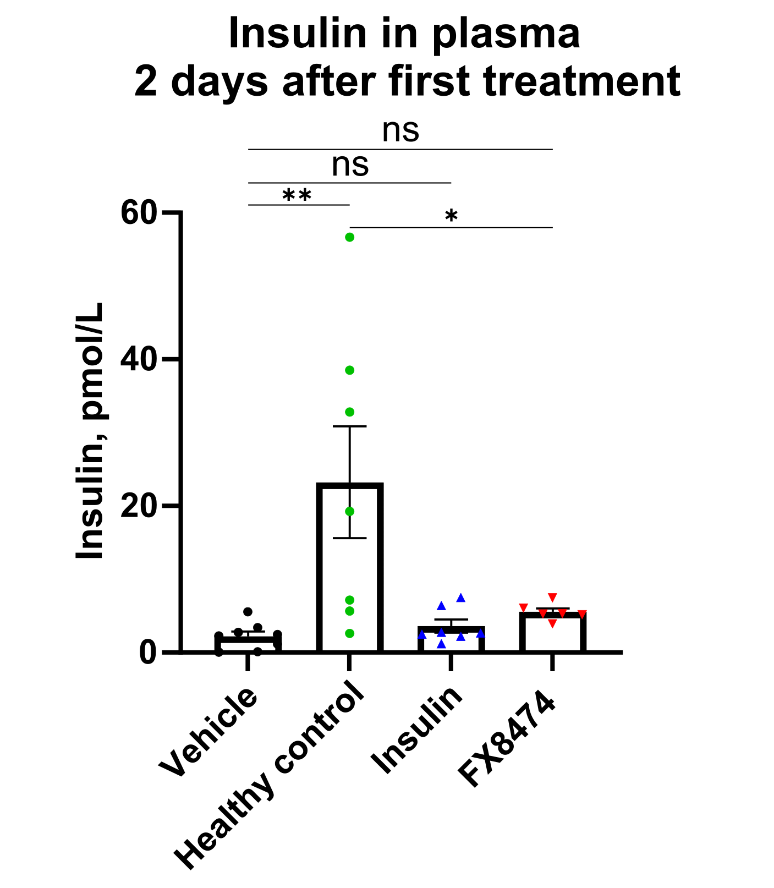
**

**Figure S3. Insulin levels in plasma 2 days after the first treatment.** One-way ANOVA was performed to determine statistical significance. Asterisks indicate statistical significance compared to vehicle-treated group.ns not significant; * p<0,5; ** p<0,01. Mean values with SD are plotted. N=6-7.


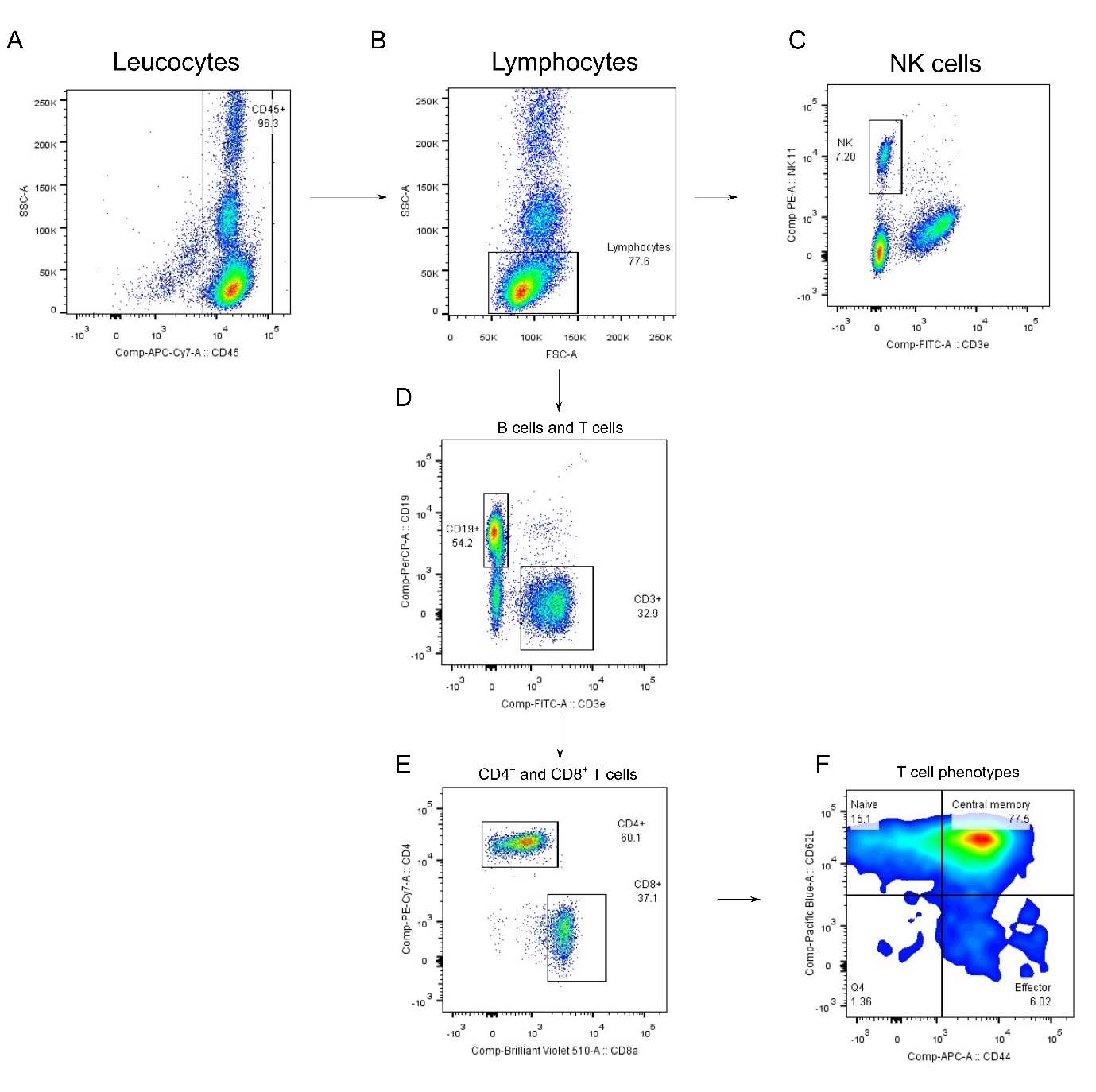


**Figure S4. Immunophenotyping gating strategy.** CD45+ gating was used to determine leukocyte, of which the lymphocyte population was determined. The Lymphocyte population was used to gate the NK cells (NK1.1+ CD3-), B cells (CD19+ CD3-) and T cells (CD3+). The T cell population was used to gate for CD4+ cells (CD4+, CD8-) and CD8+ cells (CD8+ CD4-). From each of the T cell subset their phenotypes were determined: Naive (CD62L+, CD44-), Central memory (CD62L+, CD44+) and effector memory (CD62-, CD44+).


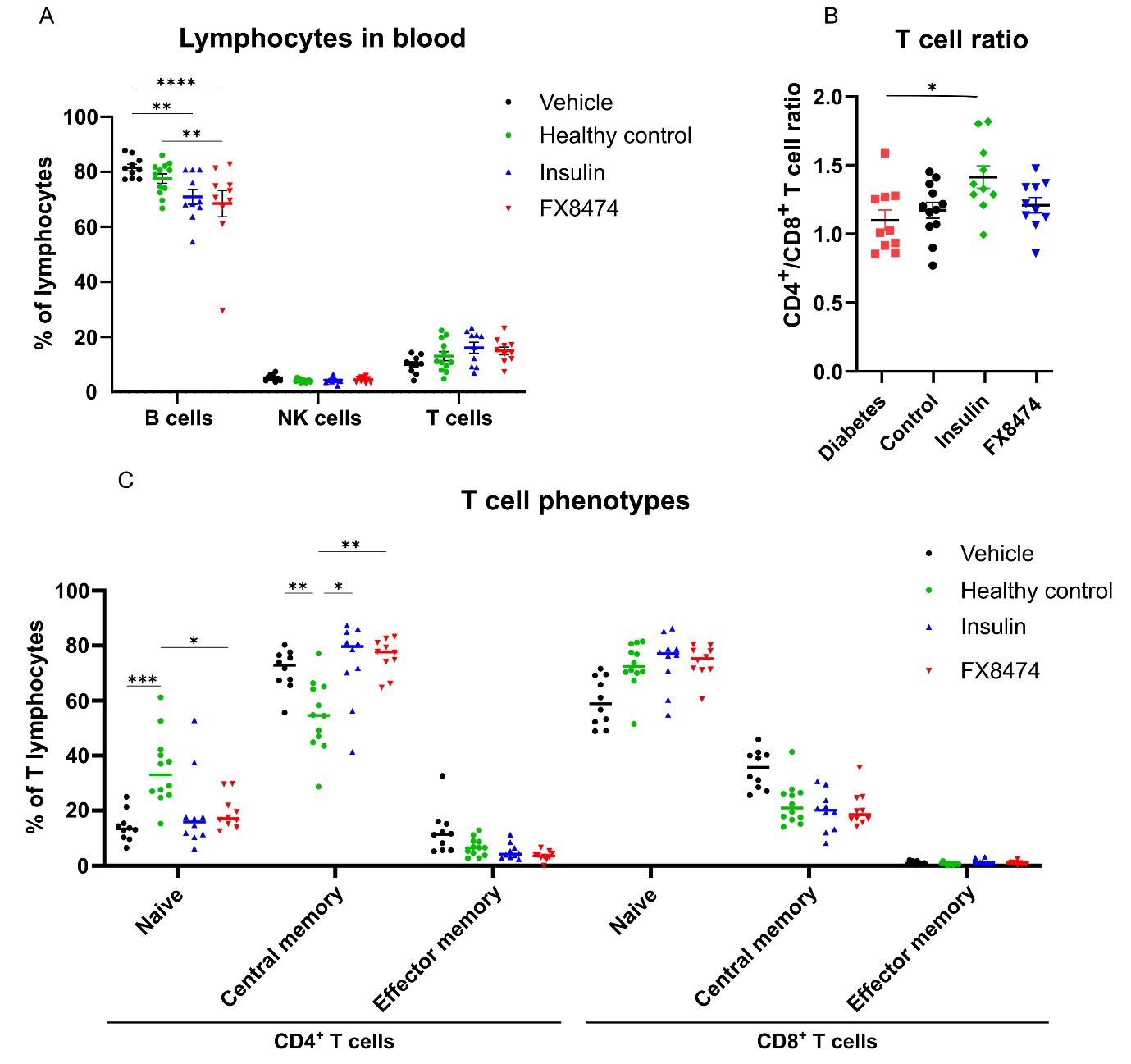



**Figure S5. Immunophenotyping from blood.** Whole blood was collected 2 days after the first treatment cycle and immunophenotyping was performed. **A)** Lymphocyte populations in blood. Statistical significance was determined by Two-Way ANOVA with Tukey’s multiple comparison test. Only statistically significant differences are shown. **B)** CD4^+^/CD8^+^ T cell ratios. Statistical significance was determined by One-way ANOVA with multiple comparisons. **C)** Phenotypes of CD4^+^ and CD8^+^ T cells. Statistical significance was determined by Two-Way ANOVA with Tukey’s multiple comparison test. * p<0,05, ** p<0,01. N= 6. Only statistically significant differences are shown.


Supplement: Supplementary file 3 [file Supplementaryfile1.docx]
